# Gait Phase Classification and Assist Torque Prediction for a Lower Limb Exoskeleton System Using Kernel Recursive Least-Squares Method

**DOI:** 10.3390/s19245449

**Published:** 2019-12-10

**Authors:** Yue Ma, Xinyu Wu, Can Wang, Zhengkun Yi, Guoyuan Liang

**Affiliations:** 1 Guangdong Provincial Key Laboratory of Robotics and Intelligent System, Shenzhen Institutes of Advanced Technology, Chinese Academy of Sciences, Shenzhen 518055, China; yue.ma@siat.ac.cn (Y.M.); xy.wu@siat.ac.cn (X.W.); zk.yi@siat.ac.cn (Z.Y.); gy.liang@siat.ac.cn (G.L.); 2 Shenzhen College of Advanced Technology, University of Chinese Academy of Sciences, Beijing 100049, China; 3 CAS Key Laboratory of Human-Machine Intelligence-Synergy Systems, Shenzhen Institutes of Advanced Technology, Shenzhen 518055, China; 4 SIAT Branch, Shenzhen Institute of Artificial Intelligence and Robotics for Society, Shenzhen 518055, China

**Keywords:** gait phase classification, exoskeleton, KRLS, MLPNN, SVM

## Abstract

The gait phase classification method is a key technique to control an exoskeleton robot. Different people have different gait features while wearing an exoskeleton robot due to the gap between the exoskeleton and the wearer and their operation habits, such as the correspondence between the joint angle and the moment at which the foot contacts the ground, the amplitude of the joint angle and others. In order to enhance the performance of the gait phase classification in an exoskeleton robot using only the angle of hip and knee joints, a kernel recursive least-squares (KRLS) algorithm is introduced to build a gait phase classification model. We also build an assist torque predictor based on the KRLS algorithm in this work considering the adaptation of unique gait features. In this paper, we evaluate the classification performance of the KRLS model by comparing with two other commonly used gait recognition methods—the multi-layer perceptron neural network (MLPNN) method and the support vector machine (SVM) algorithm. In this experiment, the training and testing datasets for the models built by KRLS, MLPNN and SVM were collected from 10 healthy volunteers. The gait data are collected from the exoskeleton robot that we designed rather than collected from the human body. These data depict the human-robot coupling gait that includes unique gait features. The KRLS classification results are in average 3% higher than MLPNN and SVM. The testing average accuracy of KRLS is about 86%. The prediction results of KRLS are twice as good as MLPNN in assist torque prediction experiments. The KRLS performs in a good, stable, and robust way and shows model generalization abilities.

## 1. Introduction

Wearable exoskeletons have recently been extensively researched [1,2]. Regarding medical applications, the lower limb exoskeleton has widely been researched, because it can assist elderly people or those suffering from muscle weakness to walk easily and also help people suffering from strokes or paraplegics to walk again. In past decades, numerous exoskeletons were designed such as the hybrid assistive limb (HAL) [3], EKSO [4], ReWalk [5], Indego [6] and brain-machine-interface (BMI)-controlled rehabilitation exoskeletons [7,8,9].

The exoskeleton robots work in different modes under different gait phases. Gait phase classifiers could be used to control walking-assistive devices for people [10]. The method of providing the specific joint torque in a specific gait phase is widely used for controlling exoskeleton robots. The applications of gait phase classifier can be found in exoskeletons [11], smart prostheses [12] and functional electronic simulation (FES) [13,14]. Based on the existing research results, the gait phase classifiers often use single-type sensors or a combination of multiple types of sensors, such as the angular velocity, attitude, force, electromyograph (EMG), IMU, camera and so on. However, the fewer sensors we use the easier we control the exoskeleton.

This paper mainly focuses on detecting the gait phase of a human-exoskeleton coupling system using only joint angular sensors considering the gait differences between the wearers. Different people have different gait features while wearing exoskeleton robot due to the gap between the exoskeleton and the wearer and the operation habits, such as the correspondence between the joint angle and the moment at which the foot contacts the ground, the amplitude of the joint angle and others. These differences are analyzed in detail in Section 4.1. We define these user-dependent features as the unique feature of one wearer. At the same time, the gaits have some general features, such as the angle of hip joints which have one peak and one valley in one gait cycle, nearby the peak angle of the hip and knee joints correspond to the switch of different gait phases. The gait phase can be obtained by the optimized joint angle threshold based on the general features. However, the present gait phase classifiers concentrate on utilizing the general features of the gait and omitting the unique feature when wearing the exoskeleton robot. Hence, we will investigate whether the unique gait feature is relevant to the decrease in the gait phase classification accuracy or to the performance of predicting the assist torque of the exoskeleton robot.

In this work, we adopt the KRLS algorithm to construct a gait phase classifier. There are two main advantages of using the KRLS algorithm to design the classifier. On the one hand, the computational complexity of the KRLS algorithm is low and it can be deployed in real-time control loops. On the other hand, the algorithm could update the classifier model with online changing data. In other words, the KRLS-based classifier model could adaptively obtain features of different data sets, which means that the KRLS-based classifier model has the ability to learn specific gait characteristics from the particular wearer.

To validate the performance of the KRLS based gait phase classifier, two classical gait phase recognition algorithms were used for comparison—SVM and MLPNN. To obtain the optimal parameter of the SVM algorithm, the particle swarm optimization (PSO) algorithms was applied. To validate the adaptation abilities of the KRLS algorithm, we designed the assist torque prediction experiment.

There are two main contributions of this work. First, to the best of our knowledge, we were the first to apply KRLS to gait phase classification and assist torque prediction to control the exoskeleton robot. Second, we found that the unique gait feature has an effect on the generalization and robustness of the gait phase classifier. Therefore, the online adaptation ability is needed for the gait phase classification or assist torque prediction.

The remainder of this paper is organized as follows. We introduce and discuss the related works in Section 2. In Section 3, we introduce the data acquisition platform, data source and data processing at first, then KRLS, MLPNN, and SVM algorithms are described for constructing gait phase classifiers and assist torque predictors. Experimental processes and results are comprehensively described in Section 4. We discuss the results in Section 5. Section 6 concludes this work and the future work.

## 2. Related Work

Many researchers used the gait phase classification information to control the lower limb exoskeleton. Kazerooni [11] presented a hybrid control scheme to control the Berkeley lower extremity exoskeleton (BLEEX). In their work, the walking gait cycle was divided into two phases, swing phase and stance phase. Meanwhile, two controllers with different control schemes were adopted. A sensitivity amplification controller was used for the swing leg, while a position control was used for the stance leg. The position controller can be implemented in many ways [15,16]. Li [17] utilized a finite state machine to estimate the current gait period of the wearer among the six major periods. After that, an impedance-based controller was adopted to provide functional gait assistance in each gait period. Chen [18] presented a novel gait phase-based control strategy for a portable knee-ankle-foot robot in which the assistive torque was only provided for selected gait phases.

Thanks to recent advances in wearable sensor technologies such as goniometers, accelerometers, gyroscopes, inertial measurement units [19], force sensing registers (FSRs) [20,21,22,23,24] and sensor fusion technology [25], various sensor-based gait phase classification methods were developed in exoskeleton robots in which different sensors were used to distinguish the gait phase.

Jung [10] utilized two types of artificial neural networks to classify the gait phase using signals from the angular sensors in the lower limb exoskeleton. The classifiers use the angle of each lower limb and the angular velocities of joints as input to obtain the gait phase. Guo [26] proposed a C4.5 decision tree to identify the gait phase. Joint angles, its angular velocity and the contact force of the foot were measured. Next, five phases of the gait were identified by the C4.5 decision tree algorithm. Heo [27] used recurrent neural networks (RNN) to categorize a gait cycle into a swing phase and a stance phase. The input data of the RNN was composed of joint angles alongside its velocities and a back propagation algorithm was used to train the network. Kong [28] noted that, although ground contact forces (GCF) provide detailed information to detect gait phase, the idea of simply using a threshold to predict the gait phase could not achieve satisfactory results. Therefore, a fuzzy logic method was applied to obtain a smooth and continuous gait phases detection. Mannini [29,30] used hidden Markov models (HMMs) to estimate four gait phases using the signals from the gyroscope mounted on the foot instep. Another work [31] adopted linear discriminant analysis (LDA) to separate eight different phases of a gait cycle using the electromyographic (EMG) signals of the lower limb. Zheng [32] presented a walking-speed-adaptive estimation method of the gait phase through novel noncontact capacitive sensors and adaptive oscillators. These were aimed at real-time gait phase estimation tasks. In Reference [33], the MLPNN algorithm was used to achieve gait phase recognition for lower limb exoskeletons using only joint angular sensors. Nevertheless, all of these studies neglected the unique feature of different peoples’ gaits.

The KRLS algorithm was used in the load forecasting of power systems for online, real-time property [34]. In Reference [35,36], Pang employed KRLS in an FPGA to implement online non-linear regression. Inspired by the real-time and nonlinear properties of KRLS, this work employs KRLS to address the gait phase classification problem in the exoskeleton robot.

## 3. Materials and Methods

In this section, the data acquisition platform is described, then the data source is described. After that, we show how we divided the gait phase and how to define the assist torque. Finally, the gait phase classification and prediction model is introduced.

### 3.1. Data Acquisition Platform

**Figure 1:**
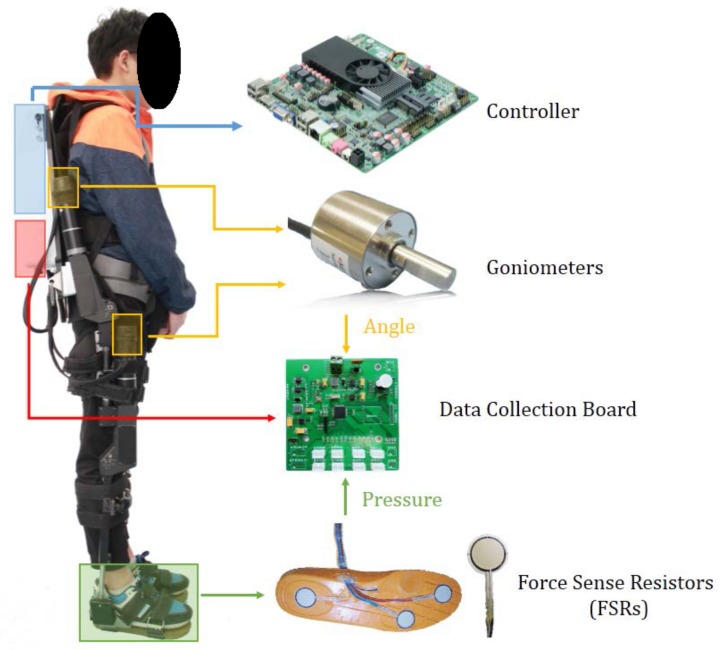
The control and sensing system of the Shenzhen Institutes of Advanced Technology (SIAT) lower limb exoskeleton robot.

The data acquisition platform is shown in Figure 1. There are two kinds of sensors in this exoskeleton robot, the goniometers and the force sensing resistors. The lower limb exoskeleton designed by the Shenzhen Institutes of Advanced Technology (SIAT), Chinese Academy of Science called the SIAT exoskeleton was used in our experiments [37]. This exoskeleton was designed with the aim of helping the paraplegic patients to walk again normally or in the rehabilitation training for people suffering from lower limb injuries, such as accidents or strokes.

Since the first generation exoskeleton designed in 2012, a third version of the SIAT exoskeleton has been redesigned. After several improvement proposals in recent years, the newest SIAT exoskeleton has a more reasonable mechanism structure with lighter weight (27 kg), which means that users would feel more comfortable when wearing the exoskeleton. Currently, our exoskeleton has three joints in each leg, including ankle joint, knee joint and hip joint. Overall, there are three degrees of freedom (DoFs) in each leg of the exoskeleton and a total of six joint DoFs for the entire exoskeleton. The three joints in each leg can all produce the flexion/extension (F/E) motion to produce movement in fore-and-aft directions. The distributions of DoFs and actuator types of joint are shown in Table 1. It should be mentioned that the knee and hip joints are active and driven by the DC motors, while the ankle joint is passive with compliant springs, so as to reduce the impact and shock when contacting the ground. To guarantee that the joints can provide enough torque for the entire exoskeleton and the wearer, we chose the 200 watt RE 50 maxon DC motor. Additionally, the length of the thigh and shank in each leg of the exoskeleton can be adjusted to fit the different height of wearers. The main characteristics of SIAT Exoskeleton robot are shown in Table 2.

In order to obtain the state information of the SIAT exoskeleton and use it for the controller to decide the following movement, goniometers are installed in the hip and knee joints to collect their angle information. The used goniometers are WDG-AM23-360, which can record the absolute angle range from 0 to 360 with 12-bit resolution. This means that angular measurement precision can be as high as 0.087°.

Meanwhile, three force sense resistors (FSRs) are contained in an insole place in the shoes to detect the relationship between the foot and the ground. As shown in Figure 1, three FSRs are placed on the heel, middle and toe. The FSR 402 sensors equipped in our exoskeleton are produced by Interlink Electronics. FSR is a polymer thick film (PTF), shown in the bottom of Figure 1, whose resistance decreases when the force that is applied to the active surface increases. As the wearer walks in a gait cycle, the output voltage changes corresponding to the variation resistance of the FSR.

The SIAT exoskeleton has two working modes—power-assist mode and zero-torque mode—which are used for different condition. The clutches are designed on each joint to switch between the two modes [33]. The power-assist mode offers enough torque to let the wearers walk in the predefined trajectory with the smart crutches to maintain balance. In the zero-torque mode, the wearers can walk following their own will and the exoskeleton offers torque as needed to follow the pilot. The zero-torque mode is used for collecting gait data with angle sensors described above.

### 3.2. Data Source

The gait data we collect comes from 10 healthy volunteers who never suffered from gait dysfunctions. The heights of the volunteers varied between 161 cm to 177 cm and their weights ranged from 53 kg to 70 kg. Before data collection, all the participants understood the purpose and the procedure of the data collection. The detailed information of the volunteers is described in Table 3.

Before gait data collection, the shank and thigh of the exoskeleton were adjusted to ensure that the joint axes of the exoskeleton and the wearer are aligned. Each volunteer was then asked to wear the SIAT exoskeleton robot set in zero-torque mode and to walk in any comfortable way for a while. After the above adjustment, we started to collect gait data. In this procedure, volunteers were asked to walk 5 m on a flat surface with their normal walking speed while a host computer recorded the data from the goniometers and FSR. Each volunteer repeated the above procedure 5 trials. Then start to record data from another subject.

**Figure 2:**
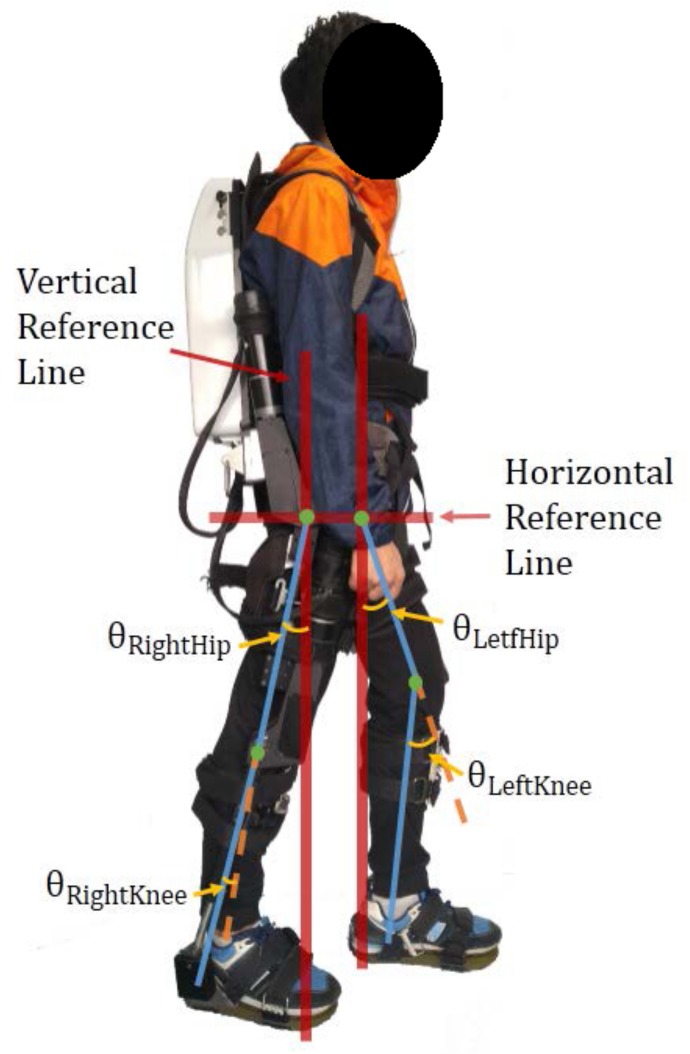
The definitions of the joint angle deviating from standard standing.

Figure 2 shows how to define the joint angles: the angle of the hip is the deviation from the standing baseline to thigh baseline, while the angle of the knee is the deviation from the thigh baseline to shank baseline. The direction of the hip extension is defined as negative, while the direction of the knee flexion is defined as positive.

**Figure 3:**
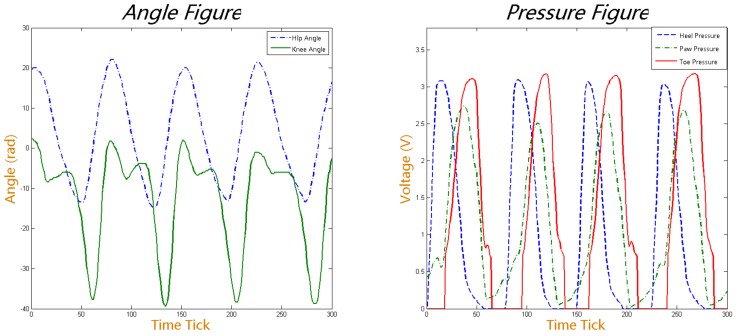
A segment of the collected joint angle data (**left**) and force sense resistors (FSR) data (**right**). In the left figure, the blue dashed line represents the left hip joint angle and the green solid line represents the left knee joint angle. In the right figure, the red solid line represents toe pressure, the green dashed line represents heel pressure and the blue dotted line represents 4/5th metatarsal pressure.

Finally, we obtained angle information from the knee and hip joints and FSRs data from the feet with 50 Hz sampling frequency. One segmentation of angle information of the hip and knee joints and FSRs of the foot are shown in Figure 3.

The data collected while the subjects stopped walking and were standing still are removed to ensure all the data used in our experiments is walking gait data. Fifty series of gait data have been collected with each subject having different walking phase, step length, step height and walking cadence to ensure the volunteer operate the exoskeleton robot as their will. Finally, we obtained a total of 24,408 samples from the collected data that are to be used in our later experiments. Every sample is composed of five dimensions, that are the hip joint angle, knee joint angle, heel pressure, paw pressure, and toe pressure. The FSR data are to be used to confirm the current gait phase, which is described in detail later.

### 3.3. Gait Phase Division and Assist Torque Definition

The gait phase division and the assist torque definition are used to generate the label of the gait data in the classification experiment and the prediction experiment, respectively.

#### 3.3.1. Gait Phase Division

Rancho Los Amigos (RLA) terminology is often used as the standard way to divide gait phase into 8 phases, this has become the mainstream since the late 1980s [38]. RLA terminology divides gait phases into initial contact, loading response, mid-stance, terminal stance, pre-swing, initial swing, mid-swing and terminal swing.

In this work, our aim is to enhance the rehabilitation of hemiplegic patients who have only one dysfunctional leg. This could be done by controlling the exoskeleton robot to assist the disabled leg using the coupling relationship between the two legs. The gait phase information from the healthy leg is used to estimate the walking phase of the disabled leg and the different manually adjusted torques are used to assist the disabled leg in different walking phases. Hence, all we need to do is to correctly identify the gait phase of the healthy leg.

**Figure 4:**
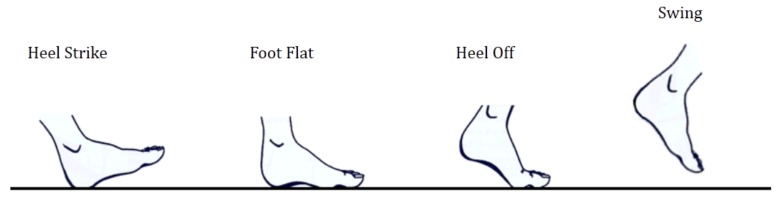
Relationship between the foot and ground.

Motion information of the healthy leg is described by the RLA stance phase states. When we decouple the phase relationship of the left and right legs in RLA gait terms, it is easy to find that the states of one leg phase can be classified into 4 phases based on the relationship between the foot and the ground which can be defined as—heel strike (HS), foot flat (FF), heel off (HO) and swing (SW) [39] as shown in Figure 4. This relationship in stance foot (STF) and swing foot (SWF) can represent one-leg phase information. In this paper, we used HS, FF, HO, and SW to divide the gait phase.

The one leg gait phase could be divided based on the FSR data and used as the switching value. Therefore, the label of HS, FF, HO and SW should be calculated before gait classification. Each FSR and series of walking has a different threshold, the thresholds could be separately calculated using the following equation

(1) $$\begin{array}{c} \begin{array}{c} D_{threshold}=\frac{y_{max}+2\left(1-\sigma \right)y_{min}}{1+2(1-\sigma )}, \end{array} \end{array}$$

where $D_{threshold}$ is an FSR threshold of one series of the walking gait, $y_{max}$ is the maximum value of an FSR series, $y_{min}$ is the minimum value of the FSR series and σ is the coefficient that represents the percentage of FSR threshold. In these experiments, σ is set to 50%. Therefore, we can obtain three thresholds for heel FSR, sole FSR and toe FSR. If the voltage of the FSR data is larger than the corresponding threshold, then the state of this FSR is high, otherwise it is low. After obtaining the threshold of each FSR in each series, a truth table is created to divide the gait phase, as shown in Table 4 [37].

#### 3.3.2. Assist Torque Definition

The hip joint assist torque is a set of continuous smooth curves and it is defined based on the gait phase. According to the clinical gait analysis (CGA), from the HS to HO of one leg, the corresponding hip joint performs a flexion torque, while from the HO to SW of one leg, the corresponding hip joint performs an extension torque. Hence, the contralateral leg is the opposite. We define the extension torque to be positive and the flexion torque to be negative. At the middle of HS and FF of the healthy leg, the assist torque is defined as 5 Nm. At the rising edge of FF to HO of the healthy leg, the assist torque is defined as 0 Nm. At the middle of HO and SW of the healthy leg, the assist torque is defined as −5 Nm. Finally, at the falling edge of SW to HS of the healthy leg, the assist torque is defined as 0 Nm. The spline interpolation method in MATLAB is employed to get the continuous assist torque.

### 3.4. Gait Phase Classification and Prediction Model

In this paper, the KRLS, MLPNN and SVM algorithms are employed to classify the gait phase. The particle swarm optimization (PSO) is used to optimize the parameters of SVM. KRLS and MLPNN are also employed in the regression of the assist joint torque of the exoskeleton robot.

In the gait phase classification experiment, a sliding window for five sample points is employed. The input of classifier is defined as

$$\begin{array}{c} \left[x_{k}(t)\;x_{k}(t-1)\;x_{k}(t-2)\;x_{k}(t-3)\;x_{k}(t-4\right)\\ x_{h}(t)\;x_{h}(t-1)\;x_{h}(t-2)\;x_{h}(t-3)\;x_{h}(t-4)], \end{array}$$

where $x_{k}\left(t\right)$ and $x_{h}\left(t\right)$ are the angles of the knee and hip joints at time *t*, respectively. The output of these classifiers is a discrete gait phase.

In the assist torque prediction experiment, a sliding window for five sample points is also employed. The input of the predictor is defined as

$$\begin{array}{r} \left[x_{k}(t)\;x_{k}(t-1)\;x_{k}(t-2)\;x_{k}(t-3)\;x_{k}(t-4\right)\\ x_{h}(t)\;x_{h}(t-1)\;x_{h}(t-2)\;x_{h}(t-3)\;x_{h}(t-4)\\ y_{T}(t-1)\;y_{T}(t-2)\;y_{T}(t-3)\;y_{T}(t-4)\;y_{T}(t-5)], \end{array}$$

where $x_{k}\left(t\right)$ and $x_{h}\left(t\right)$ are the angles of the knee and hip joints at time *t* and $y_{T}\left(t\right)$ is the target assist torque at time *t*. The target assist torque at times t−1, t−2, t−3, t−4, t−5 is employed as the input in the prediction task. The output of these predictors is a continuous assist torque.

#### 3.4.1. The Kernel Recursive Least-Squares Algorithm

The KRLS algorithm [40] is the nonlinear version of the recursive least-squares (RLS) algorithm, which could change the structure and parameter of the classification model adaptively when using varying data. Using a sequential sparse process, the KRLS algorithm was allowed to run in real time. Here, we introduce the algorithm.

Given a recorded set of data

(2) $$\begin{array}{c} \begin{array}{c} Z^{t}=\left(x_{1},y_{1}\right),\left(x_{2},y_{2}\right),\ldots ,\left(x_{t},y_{t}\right), \end{array} \end{array}$$

where *Z* is the data set of the sampling point of the walking data, *t* is the time step, $x_{i}$ is the joint angle at time step *i*, and $y_{i}$ is the corresponding gait phase.

The simple form of RLS minimizes the sum of squared errors at each time step *t* and could be calculated as follows

(3) $$\begin{array}{c} L\left(w\right)=\sum_{i=1}^{t}{\left(f\left(x_{i}\right)-y_{i}\right)}^{2}={∥\Phi_{t}^{T}w-y_{t}∥}^{2}, \end{array}$$

(4) $$\begin{array}{c} w_{t}=\sum_{i=1}^{t}\alpha_{i}\phi \left(x_{i}\right)=\Phi_{t}^{T}\alpha , \end{array}$$

(5) $$\begin{array}{c} L\left(\alpha \right)={∥K_{t}\alpha -y_{t}∥}^{2}, \end{array}$$

where ϕ is a mapping: χ⟶H from the original space to the Hilbert space, $\alpha ={\left(\alpha_{1},\ldots ,\alpha_{t}\right)}^{T}$, $\Phi_{t}=\left[\phi \left(x_{1}\right),\phi \left(x_{2}\right),\ldots ,\phi \left(x_{t}\right)\right]$, substituting into Equation (3) we have Equation (5). Where $K_{t}=\left[\Phi_{t}^{T}\Phi_{t}\right]$ and $K_{ij}=\langle \phi \left(x_{1}\right),\phi \left(x_{2}\right)\rangle$, $\langle \cdot ,\cdot \rangle$ is the inner product in the feature space can be expressed in terms of the kernel function. In this paper, the Gaussian kernel is used $\alpha_{t}=K_{t}^{+}y_{t}$.

Usually, we minimize Equation (3) w.r.t. *w*, to obtain

(6) $$w_{t}=\min_{w}{∥\Phi_{t}^{T}w-y_{t}∥}^{2}={\left(\Phi_{t}^{T}\right)}^{+}y_{t},$$

where ${\left(\cdot \right)}^{+}$ denotes the pseudo-inverse. Fortunately, it can be easily verified that the optimal weight vector can be expressed as in Equation (4) by simply adding some vector $\bar{w}$ that is orthogonal to $\phi (x_{i})$ to $w_{t}$ and substituting into Equation (3).

To reduce the space and time required for the calculation and increase adaptive ability, we use the sparse method and have

(7) $$w_{t}=\Phi_{t}\alpha_{t}\approx \tilde{\Phi_{t}}A_{t}^{T}\alpha_{t}=\tilde{\Phi_{t}}\tilde{\alpha_{t}},$$

where $\tilde{\alpha_{t}}$ is a vector of *m* “reduced” coefficients that can be used in the online scenario.

Here we use the one-versus-all strategy to distinguish one gait phase from another. Therefore, for *n* classification tasks, we have *n* classifications and the predicated class is expressed as

(8) $$y=\max_{c}f_{c}\left(x_{i}\right),$$

where c∈1,…,n represents the corresponding gait phase class, $x_{i}$ is the input vector, and $f_{c}$ represents the *c*-th classifier.

#### 3.4.2. Multi-Layer Perceptron Neural Network Method

Neural networks are often used in gait phase estimation or regression as a normal method. The multi-layer perceptron neural network (MLPNN) consists of an input layer, one or more hidden layers and an output layer. It is a commonly used type of neural networks and has many applications in various disciplines. Prior research proved that 3-layer forward neural networks could approach any multivariate nonlinear functions when the used activation function is sigmoid.

The relationship between the input layer and hidden layer can be formulated as

(9) $$\begin{array}{c} h=f_{1}\left(W_{1}x+b_{1}\right), \end{array}$$

(10) $$\begin{array}{c} y=f_{2}\left(W_{2}h+b_{2}\right), \end{array}$$

where *x* is the input feature vector, $W_{1}$ and $W_{2}$ are weight matrices, $b_{1}$ and $b_{2}$ are bias vectors, and $f_{1}$ and $f_{2}$ are sigmoid functions. The weight matrices and bias vectors are optimized using back propagation algorithms.

Two hidden layers are employed in the MLPNN. The activation function is sigmoid and the maximum number of iterations is set to 1000. The number of neurons is set to 30 in data set analysis experiment.

#### 3.4.3. Support Vector Machine

Support vector machines (SVM) have the advantages of low cost and low generalization error, and are widely used in practical problems. We use the spatial features from walking data to train an SVM classifier to distinguish different gait phases.

The basic SVM [41,42] attempts to find a linear boundary that separates data from two classes and tries to orient the boundary so as to maximize the distance between the boundary and the nearest data points for each class. Given a dataset consisting of *m* pairs of data. $D=\{\left(x_{1},y_{1}\right),\left(x_{2},y_{2}\right),\ldots ,\left(x_{m},y_{m}\right)\}$ where the indicator $y_{i}\in \left\{‒1,+1\right\}$, the SVM will construct a boundary that can be expressed as

(11) $$\begin{array}{c} \begin{array}{c} f\left(x\right)=w^{T}x+b=0, \end{array} \end{array}$$

optimization problem

$$\begin{array}{cc} \min_{w,b} & \left(\frac{1}{2}w^{T}w+C\sum_{i=1}^{m}\xi_{i}\right)=0,\\ s.t. & \xi_{i}\geq 0,\\ & y_{i}\left(w^{T}x_{i}+b\right)\geq 1-\xi_{i},i=1,2,\ldots ,m, \end{array}$$

where *C* controls the trade-off between the model complexity and empirical risk.

Generally, we obtain linear boundaries that achieve a better training-class separation in this enlarged space and that are equivalent to nonlinear boundaries in the original space. Here, we use *G* to represent the parameter of the Gaussian kernel function. For 4-classes classification, the one-versus-all strategy is used in the experiment as same as the method used in KRLS.

#### 3.4.4. Optimization Using Particle Swarm Optimization Algorithm

To acquire satisfactory results, we must fine tune the above-mentioned parameters *C* and *G*. Thus, we employ the PSO algorithm [43] to optimize the parameters. The PSO is an effective method in multi-objective optimization. The PSO algorithm randomly initializes a group of particles, then optimizes the parameters *C* and *G* by iterations. There are two fitness values in the update iteration: the individual fitness representing the current local optimal solution, and the global fitness indicating the global optimal solution. Here, we use the prediction success rate (PSR) produced by SVM through cross validation as the fitness values of each particle.

The particle velocity is updated as follows

(12) $$\begin{array}{c} \begin{array}{c} v_{i}=wv_{i}+c_{1}r_{1}\left(p_{i}-x_{i}\right)+c_{2}r_{2}\left(g_{i}-x_{i}\right), \end{array} \end{array}$$

where $v_{i}$ is the velocity of *i*-th particle and $v_{i}\in \left[-v_{max},v_{max}\right]$, *w* represents the inertia weight factor, $c_{1}\geq 0$ and $c_{2}\geq 0$ are learning factors and $r_{1}\in \left[0,1\right]$ and $r_{2}\in \left[0,1\right]$ are random numbers.

The position of the particle is updated by

(13) $$\begin{array}{c} \begin{array}{c} x_{i}=x_{i}+v_{i}, \end{array} \end{array}$$

where $x_{i}$ is the position of *i*-th particle in the search space.

#### 3.4.5. Evaluation Criterion

In order to compare the performances of different classifiers, we define the correct rate (CR) of the dataset as

$$\begin{array}{c} \begin{array}{c} CR=\frac{correctly\;estimated\;sample\;points}{total\;sample\;points}\times 100\%. \end{array} \end{array}$$

In order to compare the effects of different predictors, we define the evaluation criteria as

$$\begin{array}{c} \begin{array}{c} \mathrm{MSE}=10\mathrm{lg}\left(\sum_{i=1}^{N}\left({\left(y\left(i\right)-y_{p}\left(i\right)\right)}^{2}\right)\right) \end{array} \end{array}$$

where *N* is the size of the test set, y(i) and $y_{p}\left(i\right)$ are the target value and prediction value, respectively. The smaller the mean squared error (MSE), the smaller the error between the predicted value and the true value, and vice versa.

## 4. Results

In this section, we first analyze the collected dataset, then we analyze the effect of using the unique feature of gaits on the performance of the MLPNN classifier. After that, we compare the classification results of KRLS, MLPNN and SVM without considering the effects of unique feature. Finally, considering the effects of unique feature, the assistive joint torque curves are adaptively predicted by KRLS. Simultaneously, the predicted results are compared to that of MLPNN that do not adaptively adjust the weight matrix in the test set.

### 4.1. The Results of Data Set Analysis

**Figure 5:**
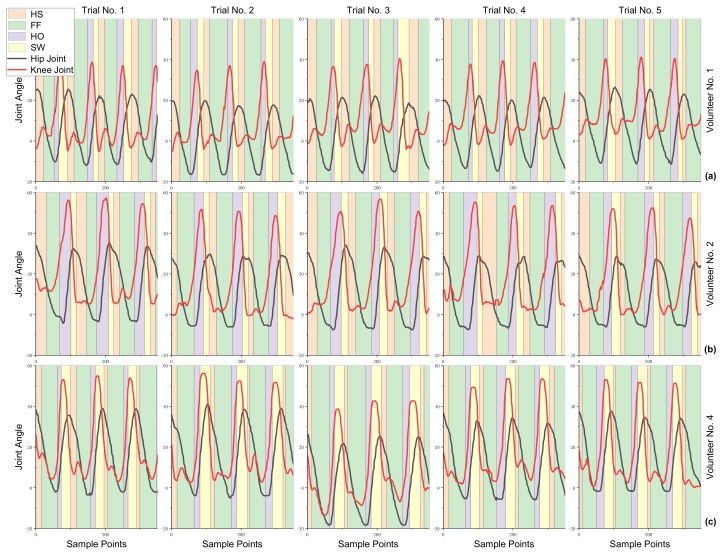
The data processing results of volunteer No. 1, No.2 and No.4. (**a**) are the five trials joints angle data from volunteer No. 1. (**b**) are the five trials joints angle data from volunteer No. 2. (**c**) are the five trials joints angle data from volunteer No. 4. The Red line represent the angle of hip joint, the black line represents the angle of knee joint. The pink, green, purple, yellow rectangle represents the gait phase of HS, FF, HO and SW respectively.

**Figure 6:**
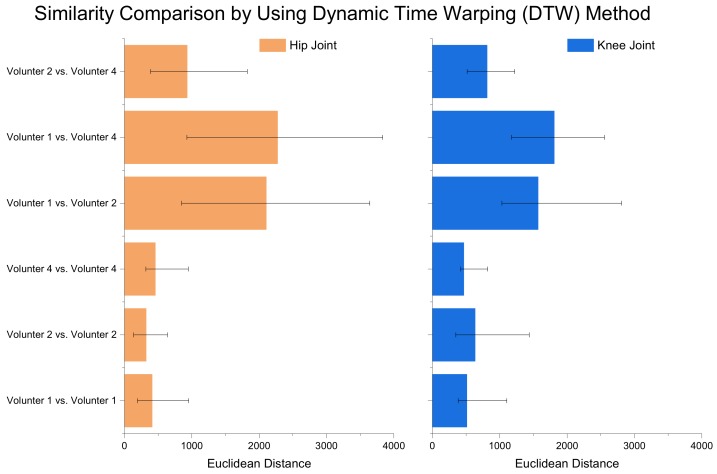
The joint angle curves similarity comparison results of volunteer No. 1, No. 2 and No. 4 by using dynamic time warping method. Each volunteer has similar joint curve shown in Volunteer No. 1 vs. Volunteer No. 1, Volunteer No. 2 vs. Volunteer No. 2 and Volunteer No. 4 vs. Volunteer No. 4. Different volunteers have different joint curves obviously, shown in Volunteer No. 1 vs. Volunteer No. 2, Volunteer No. 1 vs. Volunteer No. 4 and Volunteer No. 2 vs. Volunteer No. 4. The orange bar represent the Euclidean Distance of hip joint angle curves. The blue bar represent the Euclidean Distance of knee joint angle curves. The Matlab ’dtw’ command is used to obtain these results.

The results of joint angle data and gait phase label of the five trials done by volunteers No. 1, No. 2 and No. 4 are shown in Figure 5. Each walking gait phase is divided as discussed above. By comparing the results of each volunteer, we can see that the walking gaits in different trials are similar, and the gait phasing results are consistent. However, comparing the results of different volunteers, the gait phasing results differ from one to another. The following remarks can be seen—No. 1 has a longer FF phase shape than No. 2 and No. 4, No. 4 has the longest SW phase shape overall and the shape of HS and FF phase in No. 1 and No. 2 are similar. Hence, in this dataset, the unique gait features of different volunteers are included. By comparing the joint angles of different volunteers and the corresponding gait phase segmentation results, it can be found that different volunteers obviously have different correspondence between the joint angle and the moment at which the foot contacts the ground, as well as the amplitude of joint angle. In Figure 6, the dynamic time warping (DTW) method is employed to mathematically compare the similarity of joint angle curves. It is shown that the joint curve of different volunteers has different formations. Hence, it is difficult to accurately classify the gait phase when different people are using the exoskeleton robot as the data results shown in Figure 5 suggest.

In order to analyze the common feature of gaits in the collected data set and the unique gait feature of different volunteers, we first analyze the data consistency of the same volunteer. That is, to classify the user-dependent gait phase. The MLPNN, the commonly used method, is employed to classify the gait phase using Equations (9) and (10). The gait data of each volunteer adopts 5-fold cross-validation.

**Figure 7:**
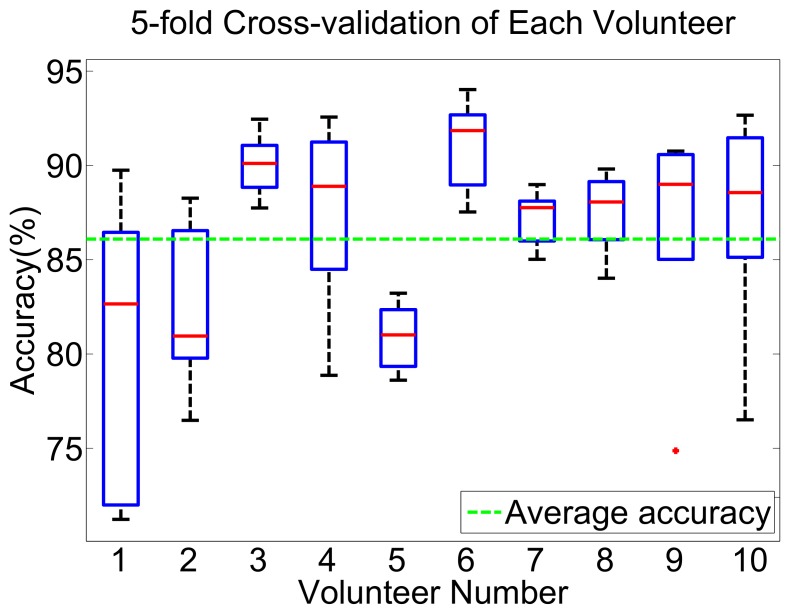
The box figure of 5-fold cross validation of each volunteer. Five trial gaits of each volunteer are 5-folded and cross validated. The green line is the average accuracy of all of these experiments.

The classification results of each volunteer are shown in Figure 7. In this figure, the volunteers No. 3, 4, 6, 7, 8, 9 and 10 have an CR over 85% and the distribution of 5-fold cross-validation results is concentrated over the average line. This suggests that the data of these volunteers have less variation and have more consistency. The results of No. 1 and 2 are more variation than the other volunteers. However, the maximum CR is near 90%, which means that the gait feature of some trial could be accurately obtained from other trials but the data of some trials do not contain enough gait features.

To avoid the disturbance of non-consistency, we use the data of volunteers No 3, 4, 6, 7, 8, 9 and 10 to validate the effects of their unique gait features and common gait features. Two groups of experiments are designed—Trial 1 (T1) and Trial 2 (T2). In T1, we randomly select one or two sets of gaits as the test set for each volunteer’s gait, that is using 70% of the gait data for the training set and 30% for the test set. In T2, we randomly select two volunteers’ gait data as the test set, using 25 trials (from 5 volunteers) of the gait data for the training set and 10 trials (from 2 volunteers) for the test set. The datasets of T1 and T2 used as an input to the MLPNN classifier that has the same configuration parameters as the cross-validation experiments. Both T1 and T2 were repeated 35 times.

**Figure 8:**
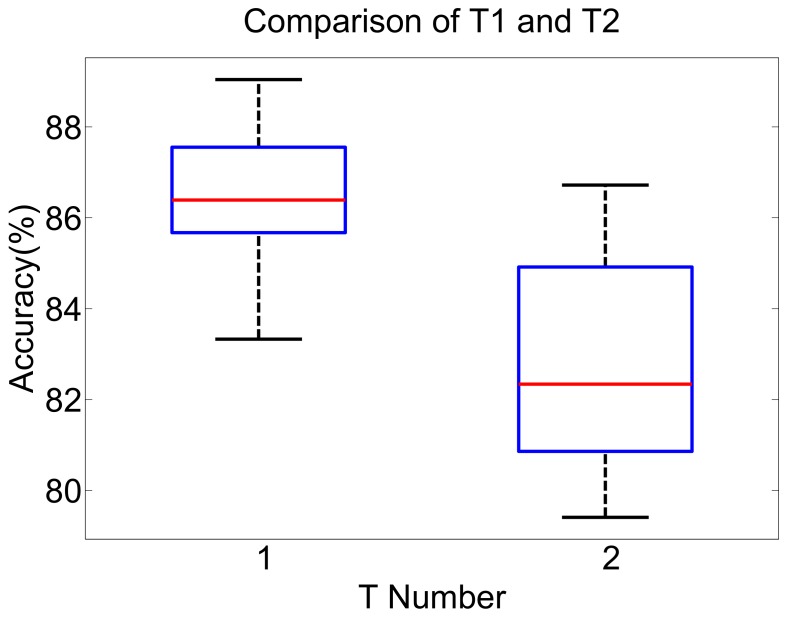
The box figure of comparison of T1 and T2. Both T1 and T2 are repeated 35 times.

The CR results of T1 and T2 are shown in Figure 8. The CR results of T1 are concentrated between 86% and 88%, while the CR results of T2 have more deviation ranging from 81% to 85%. From a qualitative view, the CR of T1 is obviously better than T2. For the sake of analysis, we separately sort the CR results of T1 and T2 and focus on the 15 results near the median value shown in Table 5. The average CR of T1 and T2 in Table 5 are 86.49% and 82.67%. The average CR of T1 is nearly 4% higher than T2. The results show that the gait features between different people are different. If the untrained gait is classified by the classifier with fixed parameters, the gait phase cannot be correctly classified.

### 4.2. Comparison and Analysis of KRLS, MLPNN and SVM

In this section, the data of 7 volunteers (No. 3, No. 4 No. 6, No. 7 No. 8, No. 9 and No. 10) are used and we show the accuracy of different parameters of all three classifiers. The training set and the test set are chosen similar to T1, but the combination of data are fixed in this part. All three classifiers use the same training and test sets. The purpose of this experiment is to verify the performance of three classification models when the data set don’t have unique gait features.

**Figure 9:**
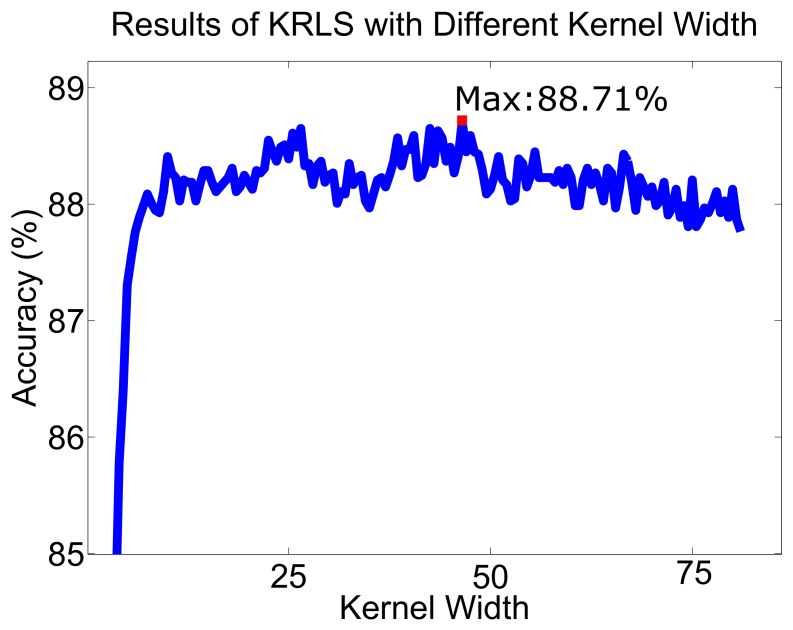
The results of KRLS with different kernel width. The maximum accuracy is 88.71% with the kernel width is 46.

The KRLS algorithm uses the kernel method, so the memory size and the width of the kernel will affect the results. In order to determine the reasonable memory size and kernel width, we try different values. Memory size is set within the range of [200 800] with 100 strides, while the kernel width is set within the range of [0.5 80.5] with 0.5 stride. Since the accuracy will stop increasing when the memory size is higher than 500 with fixed kernel width, the memory size is set to 600. The results of different kernel width values are shown in Figure 9. Figure 9 shows how the average accuracy increases when the kernel width is in the range of [0.5 46] and achieves the highest CR (88.71%) when the kernel width is 46. The accuracy decreases in the range of [46 80.5].

**Figure 10:**
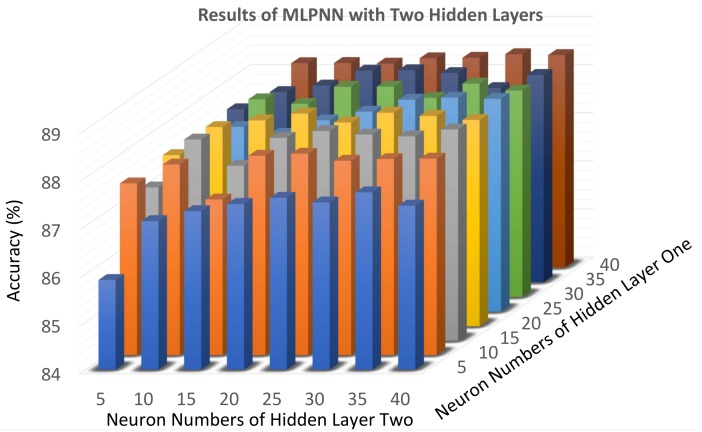
The results of different neurons in each layer. The max accuracy is 88.45%. The optimal combination of neurons is (25, 35).

Regarding MLPNN networks, we use two hidden layers. In order to obtain reliable results, we train each MLPNN model for 10 times and calculate the average accuracy to represent the performance of that model. The number of the hidden layer nodes ranges from 5 to 40 in this model with 5 strides. The activation function is sigmoid and the maximum number of iterations is set to 1000. The results are shown in Figure 10 that shows different combinations of the number of hidden layers’ nodes. When the first hidden layer has 5 nodes and the number of nodes in the second one ranges from 5 to 40, the CR is always lower than 88%. When the number of nodes ranges from 10 to 25 in the first hidden layer and from 5 to 35 in the second one, the accuracy increases as the number of neurons increases and is almost always higher than 88% and achieves the best performance (88.45%) when the first hidden layer has 25 nodes and the second one has 35.

When it comes to the SVM model, we used the PSO algorithm to determine the optimal parameters *C* and *G*. We set the population to 20, the maximum iterations to 100, learning factor $c_{1}=1.6$, $c_{2}=1.9$; inertia weight factor ω=0.6, the search range of parameter *C* is [0.001 20], the search range of parameter *G* is [0.001 30]. After optimization, The best results of *C* and *G* are 1.2 and 4.6, respectively. The maximum CR is 88.69%.

Finally, we report the best parameters of the three models based on our training and testing datasets. Using these parameters, the best CR results of the models built by KRLS, MLPNN, and SVM are 88.71%, 88.45%, and 84.22%, respectively. The best results of these classifiers using the fixed dataset are similar by selecting their optimal parameters. To test the model generalization ability of the three algorithms, five trials of data from volunteers No. 2, 3, 4, 6, 7, 8, 9, and 10 are mixed together, and a K-fold cross-validation method is used to select the training set and the test set. During cross-validation, the combined dataset is divided into K sections. Every section could be the testing set, while others are used as the training set in the cross-validation loop. Every trial is repeated 5 times and the average results are employed. Table 6, Table 7 and Table 8 provide the 3-Fold, 5-Fold, and 10-Fold CR results of cross validation, respectively. The CR result of the best performing classifier under the specific fold index is marked in bold. The CR results show that KRLS has almost the best performance under various cross-validation conditions.

Table 9, shows that the average CR of KRLS is nearly 2.33% higher than MLPNN and 2.49% higher than SVM under 3-Fold condition, about 3.62% higher than MLPNN and 3.35% higher than SVM under 5-Fold condition and finally about 3.04% higher than MLPNN and 2.98% higher than SVM under 10-Fold condition. This shows that the KRLS method has a robust and stable performance under different data conditions.

From the above comparison and analysis, we can find that the KRLS method performs better than the other two classification algorithms in gait phase classification tasks and shows a robust and stable performance in cross-validation experiments.

### 4.3. Assist Torque Prediction

Since the features of different wearers’ gaits are not similar, that is unique gait features, it is difficult for the gait phase classifiers trained using a certain dataset to accurately classify the gait phase of a wearer whose gait data are not included in the database (shown in Figure 8 and Table 5). Hence, the gait phase classifier should adaptively learn the gait features. KRLS has the ability to adaptively update its model. To validate that the KRLS method could be used in exoskeleton robot with good adaptive performance, we designed an experiment to compare the hip joint assist torque prediction task with MLPNN. The reason why we do not construct the adaptive gait phase classifier is that if the ground truth of the gait phase is known, we don’t need to build the adaptive classifier.

In order to verify the ability of adaptation, we used the data set including the gait data from all volunteers. We randomly selected the gait of 3 volunteers as the test set and used the rest as the training set. This way, it is guaranteed that the training set will not have the special gait features of the volunteers included in the test set. This data set is chosen similar to T2.

KRLS and MLPNN are employed in this section to predict the assist torque. The parameters of KRLS and MLPNN are the same as in the classification part. Since the KRLS algorithm does not require iterative optimization, the KRLS based predictor is updated adaptively in the training set and the test set. The MLPNN requires iterative optimization and the MLPNN based predictor is only updated in the training set.

**Figure 11:**
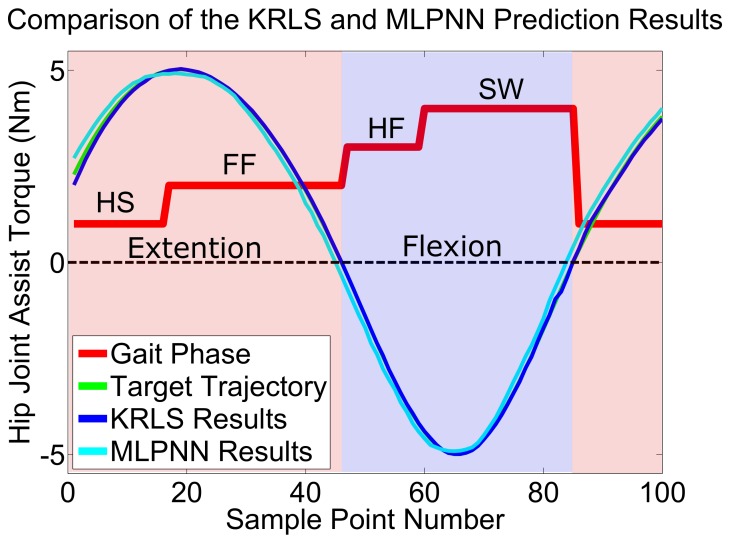
The hip joint assist toque trajectory prediction results of KRLS and MLPNN. The red line is the gait phase, the green line is the target assist torque trajectory of hip joint, the blue line is the prediction results of KRLS and the cyan line is the prediction results of MLPNN. At HS and FF phase, the assist torque help the wearer’s hip extension. At the HO and SW phase, the assist torque help the wearer’s hip flexion.

The results of assist torque prediction are shown in Table 10. The MSE of KRLS is two times smaller than MLPNN. These results show that the KRLS algorithm which has adaptive ability could predict the results quite well, even if there are unlearned unique gait features in the test set. Figure 11 shows a picked period of prediction results, in which we can find that the prediction results of MLPNN have an obvious lag and the results from KRLS basically coincide with the target assist torque trajectory.

## 5. Discussion

This paper employs three classification methods—KRLS, SVM, and MLPNN—to build recognition models of one leg gait phase and employ KRLS and MLPNN to predict assist torque of the hip joint. Angle data from goniometers on the hip and knee of a single leg were used from 10 healthy participants to train and test these models. T1 and T2 experiments are compared to show the effectiveness of unique gait features. Comparing results of KRLS, MLPNN and SVM showed that the KRLS network has the best robustness in cross-validation experiments. The KRLS network could adaptively obtain the unique gait feature of each volunteer and the results of the KRLS predictor show a great performance in the assist torque estimation task.

Using the goniometer only, the gait phase can be well identified and the sensor and control systems of the exoskeleton robot can be simplified. Our experiments show that KRLS is more robust compared with the other two algorithms and easier to obtain optimal results in the overall experiment. Additionally, the unique gait feature is useful in the gait phase-based controller to improve the robustness. Although the gait phase recognizers obtain satisfactory accuracy, there still remain unstable predictions in the case of two continuous gait phase. Therefore, some gait information is still not used to increase the accuracy.

## 6. Conclusions

Considering the unique gait features of each person, the KRLS algorithm can adaptively distinguish the gait phase based on the unique features. KRLS also has a robust performance in gait phase classification tasks compared with classical SVM and MLPNN. Compared with the gait phase classification method by plantar sensor, the proposed method uses only the joint angle information and has better adaptability to uneven surfaces. It also reduces the number of sensors and the complexity of the exoskeleton system. Compared with gait classification methods using surface EMG sensor, the proposed method is insensitive to fatigue, sweating and installation. Currently, the exoskeleton is not able to perform activities of daily living (ADL) standards and is still expensive for consumers. Furthermore, the applications of the exoskeleton robot in the medical institutes would be investigated. We expect that the exoskeleton robot will be widely used as shared equipment [44] in the architectural industry, logistics industry and many more domains. Therefore, the technologies of adaptively learning the unique gait feature would be widely applied.

We aim to increase the accuracy of gait classification in future work by using a larger data set. Research on how the exoskeleton affects normal gait of each participant is valuable to be conducted in the future to increase the accuracy of gait classification. At the same time, the online control architecture with gait classifier considering the unique gait feature for shared applications will be researched in our future work.

## Figures and Tables

**Table 1:** The joint actuator types.

Joint	DoFs	Actuator Type
Hip F/E	2	Motor-actuated
Knee F/E	2	Motor-actuated
Ankle F/E	2	Passive

**Table 2:** The main characteristics of SIAT exoskeleton.

Item	Value
Weight	27 kg
Hip joint angle range	$-10^{{}^{\circ}}$–$100^{{}^{\circ}}$
Knee joint angle range	$0^{{}^{\circ}}$–$95^{{}^{\circ}}$
Hip joint maximum torque	179.8 Nm
Knee joint maximum torque	192 Nm

**Table 3:** Volunteers information.

No. Volunteers	Weight (kg)	Height (cm)
1	65	165
2	67	175
3	53	161
4	58	175
5	61	175
6	62	170
7	55	177
8	54	170
9	58	172
10	59	176

**Table 4:** Truth table of gait phase.

Gait Phase	HS	FF	HO	SW
Heel FSR	High	High	Low	Low
Sole FSR	Low	High	Low/High	Low
Toe FSR	Low	High	High	Low

**Table 5:** 15 times CR results near the median value of T1 and T2.

T Number	T1	T2	Diff
Average	**86.49**	**82.67**	**3.82**
Max	87.46	84.24	3.22
Min	85.87	81.30	4.57

**Table 6:** 3-fold cross-validation results.

Fold Index	1	2	3
KRLS	**85.56**	**87.41**	83.5
MLPNN	80.58	84.18	**84.74**
SVM	82.74	86.27	79.98

**Table 7:** 5-fold cross-validation results.

Fold Index	1	2	3	4	5
KRLS	**87.85**	**86.38**	**85.17**	**86.23**	84.55
MLPNN	87.17	81.70	75.13	83.20	**84.89**
SVM	86.33	85.83	82.68	82.99	75.60

**Table 8:** 10-fold cross-validation results.

Fold Index	1	2	3	4	5	6	7	8	9	10
KRLS	83.52	**84.77**	**82.96**	**86.58**	**87.20**	**88.57**	**87.42**	85.85	**86.66**	**89.10**
MLPNN	**86.94**	83.83	77.90	72.31	81.23	88.00	86.64	84.70	83.72	87.01
SVM	69.06	83.93	81.76	84.69	81.83	85.86	87.03	**86.38**	84.96	87.38

**Table 9:** Average accuracy and differential between KRLS and other two classifiers.

K-Fold	3-Fold	5-Fold	10-Fold
KRLS	85.49%	86.04%	86.26%
MLPNN	83.17%	82.42%	83.23%
SVM	83.00%	82.69%	83.29%
Diff with MLPNN	**2.33**%	**3.62**%	**3.04**%
Diff with SVM	**2.49**%	**3.35**%	**2.98**%

**Table 10:** MSE of KRLS and MLPNN in assist torque prediction task.

	Trials No.	1	2	3	4	5
	MSE of KRLS	−28.05	−29.91	−28.10	−32.77	−31.61
	MSE of MLPNN	−12.26	−11.18	−13.41	−12.20	−12.26
	Diff	**−15.79**	**−18.73**	**−14.69**	**−20.57**	**−19.35**
